# The Role of Career Adaptability, the Tendency to Consider Systemic Challenges to Attain a Sustainable Development, and Hope to Improve Investments in Higher Education

**DOI:** 10.3389/fpsyg.2020.01926

**Published:** 2020-08-07

**Authors:** Ilaria Di Maggio, Maria Cristina Ginevra, Sara Santilli, Laura Nota, Salvatore Soresi

**Affiliations:** School of Psychology, University of Padua, Padua, Italy

**Keywords:** life design approach, career adaptability, sustainable career development, hope, investment in higher education

## Abstract

Based on the life design paradigm and career construction adaptation model and on recent directions from the perspective of sustainable and inclusive career guidance, the study aimed at examining the relationship between career adaptability, the tendency to consider systemic challenges to attain sustainable development, and state personal and social hope and their role on the tendency to invest in higher education. The analyses carried out involving 416 Italian high school students found that career adaptability and the tendency to consider systemic challenges in order to attain sustainable development were directly and indirectly, through state personal and social hope, related to the tendency to invest in higher education. The results obtained allowed to provide new contributions to extend results previously described by the life design approach in career development issues and provided useful suggestions for preventive career interventions.

## Introduction

During the last 30 years, many different structural and international phenomena have produced transformations and modifications in work’s features and in labor market’s demands, offering new options for workers but also putting them in front of challenges and new risks. These changes affect workers’ employment conditions but also the ideas and beliefs that young people shape about the labor market and ultimately their career trajectories. These changes may be identified at different levels (Massoudi et al., 2018).

A first aspect concerns the demographic alterations involved in these changes, due to considerable immigration flows and to the aging of populations (Massoudi et al., 2018). These demographic alterations have deeply reshaped manpower in western societies, hence incrementing workplace heterogeneity (Burke and Ng, 2006). Another aspect that has to be taken into consideration is the rapid and intense technological evolution, putting different occupational fields at risk of computerization and automation and, at the same time, promoting the need for new professional skills and profiles (Frey and Osborne, 2017). Moreover, important environmental threats and challenges can be taken into account, in terms of reduction of fossil-fuel resources and climate change, which demand various industrial models, new professional abilities, and perhaps new career models to provide a sustainable development (Massoudi et al., 2018). An additional aspect involves the economic transformations and profound pression regarding markets, as a product of globalization and neoliberal politics. These changes have been intensified by the contemporary financial crisis and the following economic recession. As a consequence, they have brought a deeply competitive and ambiguous labor market, which involves, mostly among young people, increased undignified jobs, unemployment, job insecurity, and forced career transitions for workers.

All of the aforementioned factors contributed to spread, mostly among young people, feelings of discomfort and uncertainty toward their future, the perception that the world of work is a reality full of obstacles, able to compromise the processes of educational and professional development, and, related to this, growing disinvestment in education, especially in higher education (Savickas, 2013; Pells, 2017). Higher education is essential to obtain a wide knowledge about the social, economic, and environmental international challenges that exist nowadays and to acquire the necessary skills, competences, and partnerships to launch solutions and strategies and innovative technologies to face these challenges (HEIW, 2017).

Over the last few decades, the life design paradigm (Savickas et al., 2009) has underlined the need to develop knowledge and skills to analyze non-linear causalities, complex dynamics, ecological settings, and multiple subjective contexts. Considering these assumptions, the life design approach emphasized the urgency to guide young people in choosing a satisfying career path, by considering helpful resources that can support them, especially when dealing with early repeated career transitions and with the complexity of the actual labor marker (Savickas et al., 2009).

Even if life design generally involves individual agency, the recent recognition of the current challenges that emerge from the intersection of various factors leads us to consider also the topics of social justice and sustainability, to promote and contribute to a fair and sustainable development (Guichard, 2018; García-Feijoo et al., 2020). These factors involve work scarcity, structural inequity/discrimination, and the consequent difficulties that people face during their career planning processes. Having considered all these factors, it is hard to avoid the fact that vocational guidance, career counseling, and life design need a change of pace that, according to different scholars, can only be associated with investments in inclusion and sustainability (Guichard, 2018; Nota et al., 2019).

A central core of the life design paradigm is the career construction adaptation model (Savickas et al., 2009; Savickas and Porfeli, 2012; Hirschi et al., 2015), which considers career development as a result of the combination of individual necessities and social expectations and, consequently, their adaptation to the environment (Savickas and Porfeli, 2012). The career construction adaptation model involves *adaptability resources*, a set of “psychosocial abilities that condition self-regulation in coping with the tasks, transitions, and traumas” (Hirschi et al., 2015, p. 2); *adapting responses* that are conceptualized as a set of adaptive behaviors and beliefs that people use “to address career development tasks and changing work and career conditions” (Hirschi et al., 2015, p. 2); and *adaptation results*, considered as a set of outcomes that indicate a good fit between the person and the environment. Adaptability resources predict, directly and indirectly, through a set of adapting responses, the adaptation results (Hirschi et al., 2015). A meta-analysis, based on a total of 90 studies, conducted by Rudolph et al. (2017) supported the validity of the career construction adaptation model.

It is important to take into account the new global challenges and the need to move toward a type of career development and a career planning process able to contribute to the construction of inclusive and sustainable future contexts. Specifically, we referred to global challenges related to the five areas of critical importance for humanity and the planet (people, planet, prosperity, peace, and partnership) suggested by the United Nations (2015) in the 2030 Agenda for Sustainable Development. These challenges that humankind is facing, such as climate change, water scarcity, inequality, poverty, and environmental degradation, can only be addressed by promoting sustainable development.

**FIGURE 1:**
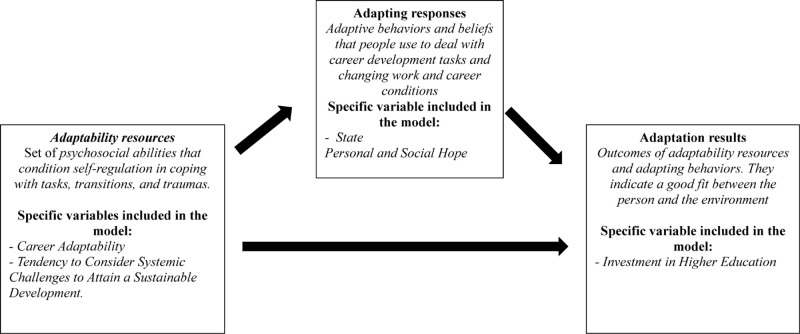
Conceptual framework based on the career construction model of adaptation (Savickas and Porfeli, 2012; Rudolph et al., 2017).

With this work, we would like to examine career adaptability, typically considered as an adaptability resource (Savickas and Porfeli, 2012; Hirschi et al., 2015; Rudolph et al., 2017). and the tendency to consider systemic challenges to attain sustainable development, as an adaptability resource to cope environmental tasks, focused on environmental and social future requests specifically related to the construction of inclusive and sustainable contexts (Nota et al., 2019, in press; García-Feijoo et al., 2020; see Figure 1).

Among the adapting responses, we took into consideration the kind of hope that the individuals feel about their situation, as analyzed, for example, by Ginevra et al. (2016) and Santilli et al. (2017). We also analyzed the kind of hope that involves social necessities, as a set of beliefs and behaviors useful to help the individual to deal with career development tasks, focusing both on the individual and on other people (Peterson and Helms, 2014; Guichard, 2018).

Finally, as concerns adaptation results, we focused on the tendency to invest in higher education (see Figure 1), because it can be relevant to contribute to a good fit between personal needs and present/future environmental expectations, also related to the need to go toward an inclusive and sustainable society (Peterson and Helms, 2014; Guichard, 2018; García-Feijoo et al., 2020).

### Investment in Higher Education

According to the data provided by Eurostat (2019), Italy is almost at the bottom of the EU table considering the number of university graduates. As a matter of fact, the percentage of Italian graduates is considered below the European average of 30%. This is unexpected because Italy has one of the highest percentages in Organization for Economic Co-operation and Development (OECD) countries (75%) of students who get a high school diploma, which allows them to have access to a university. The step to actually enroll in a university seems to be problematic: just over 70% of those who have access to a university eventually decides to enroll (Pastore, 2019). In line with these data, Tomlinson (2008) showed how young people believe that academic qualifications have a declining role as regards the construction of their career results in a labor market perceived as overcrowded, competitive, and owned by graduates. Beliefs concerning the field of university education that underestimate its benefits lead young people to be less motivated to invest in a university path. This is, most of all, due to superficial gatherings of information and frequent use of heuristic (Abbiati and Barone, 2017).

This is a worrying situation in the current context, in which higher education has become essential to prepare young people who will soon become adults and will have to face the current difficulties of the labor market and the above-mentioned global challenges (Guichard, 2018). Young people without a higher education represent one of the most fragile market segments, with the highest risk of unemployment, more difficulties in entering the labor market, and more frequent involuntary working transitions, with respect to those who reached more elevated educational levels. They are frequently employed in temporary jobs, with undignified working conditions low pay and with scarce chances of developing their skills (Wehl, 2019). Moreover, higher education is necessary to create graduates who can understand and prevent global menaces; to detect different, complex, and original solutions for these challenges; and to create a society that can contribute to well-being and satisfaction (Peterson and Helms, 2014). Moreover, these reflections represent useful stimuli for young people to promote ideas and attitudes that can help them to understand the advantages of education, to consequently motivate them to invest more in it.

### Career Adaptability

Career adaptability is a self-managing, transactional, and bending skill, useful to address developmental tasks and to handle present and future changes in career contexts. It is also useful to promote adjustment and successful shifts during the career lifetime (Rossier, 2015; Rudolph et al., 2017). It involves people’s skills to consider environmental eventualities to proactively adjust it to their necessities and values (Massoudi et al., 2018).

Career adaptability includes four problem-solving and coping strategies or resources: concern, control, curiosity, and confidence (Savickas, 2013). Concern involves being conscious about and making plans for forthcoming transitions, with a hopeful attitude with respect to the future (Santilli et al., 2017). Control involves owning the future and feeling able to make suitable career-related decisions. Curiosity concerns exploring the self and the environment. Finally, confidence concerns problem-solving skills and resources to adequately face difficulties, challenges, and impediments.

According to the career construction adaptation model, career adaptability has a predictive role on state hope, conceptualized as an adapting response. State hope is a cognitive set to start and continue actions to reach personal goals in particular times and more proximal events (Snyder et al., 1996). Different studies have supported the relationship between career adaptability and state hope: for example, Ginevra et al. (2016) observed that career adaptability partially predicted career decidedness, through the mediational role of a positive belief toward the future (hope, optimism, and lack of pessimism) and future orientation, in a sample of Italian adolescents. Moreover, Santilli et al. (2017) found that career adaptability partially predicted Italian adolescents’ life satisfaction, through a positive orientation toward the future (hope and optimism). In addition, career adaptability directly predicted life satisfaction in a sample of Swiss adolescents (Santilli et al., 2017). These findings suggested that adolescents with elevated levels of career adaptability are more capable to conceptualize different possible future scenarios. As a consequence, they present higher levels of commitment, responsibility for career choices, and career decision making and are more satisfied with their quality of life.

As regards the relation between career adaptability and investment in higher education, the meta-analysis by Rudolph et al. (2017) found that career adaptability is associated with higher levels of academic engagement. Merino-Tejedor et al. (2018) found that career adaptability fully mediated the relationship between trait emotional intelligence and academic engagement, supporting the idea that career adaptability facilitates greater investment in higher education. Akkermans et al. (2018) reported that having higher levels of career adaptability mean having a clear idea about one’s preferences and future wishes and also being able, through a motivational process, to act to detect future paths that take into consideration personal and environmental aspects.

**Hypothesis 1.1.** Career adaptability (i.e., concern, control, curiosity, and confidence) is related to state personal and social hope (see Figure 1).

**Hypothesis 1.2.** Career adaptability (i.e., concern, control, curiosity, and confidence) is related to the tendency to invest in higher education (see Figure 1).

### The Tendency to Consider Systemic Challenges to Attain a Sustainable Development

Over recent years, in the field of career guidance, researchers and professionals are increasingly directing the attention of individuals toward social challenges through both personal and systemic changes and actions (Kenny et al., 2018), supporting in this way a sustainable and inclusive development.

The concept of sustainable development regarding the social and economic development of the society requires solidarity among wealthy and poverty-stricken countries but also between the present and future generations. Said solidarity involves the safeguard of the global environment and insists on taking into consideration economic, social, and green aspects, to be understood as equivalent and reciprocally dependent. This deeply humanistic and far-sighted idea, reporting inequalities and supporting justice, should lead individuals’ daily choices; to increase universal, social, and individual welfare; and to encourage a more aware and active “presence in the world,” decent jobs, and education (Kargulowa, 2018). In this respect, people could be encouraged to be less “ego-centric”; to recognize discriminations, inequalities, barriers, and exploitations; and to act also from a professional point of view to defeat them, reduce them, and create alternatives in favor of the overall wellness of humanity and of the world we live in. They should be stimulated to be more oriented to undertake professional activities that allow them to achieve their well-being and, being inspired by less individualistic values, to contribute to the realization of inclusive and sustainable social contexts (Nota et al., 2019; García-Feijoo et al., 2020).

Many studies examined the ability to systematically analyze current social conditions and the perceived ability to reduce societal inequalities (critical conscience). Said studies suggest that critical conscience is associated to career adaptability and contributes to an investment in education, school, academic engagement, motivation, achievement, and educational/career expectations among adolescents (Duffy et al., 2016). Moreover, adolescents with vulnerabilities (i.e., Afro-American adolescents) who have a high ability to systematically analyze current social conditions and the perceived capacity to reduce societal inequalities are characterized by a feeling of hope for their vocational future. This relationship allows them to implement professional/educational choices and/or to continue an educational/professional course, despite the obstacles they experience (Diemer et al., 2006). These results tend to suggest that a higher tendency to consider global challenges could lead students to reconsider their beliefs regarding the benefits and costs associated with the pursuit of higher education. All of this, through a hopeful motivational process, could promote an increasing investment in higher education (Abbiati and Barone, 2017).

**Hypothesis 2.1.** The tendency to consider systemic challenges to attain sustainable development is related to state personal and social hope (see Figure 1).

**Hypothesis 2.2.** The tendency to consider systemic challenges to attain sustainable development is related to the tendency to invest in higher education (see Figure 1).

### State Personal and Social Hope

There are different definitions of hope that can be found in psychological literature. For example, Scioli et al. (2011) defined hope as an affective, future-directed variable, sustaining action and influencing behaviors and thoughts. Staats (1989) affirmed that hope consists of two components: a cognitive one about the expectations of a future event likelihood to happen and an affective one, connected to the expectations of good consequences or positive events. According to Snyder et al. (1996), state hope is based on two cognitions: agency thinking and pathway thinking. Agency concerns the commitment to start and keep the effort needed to reach goals in particular times and more proximal events; pathway refers to the plans needed to reach a goal. These dimensions are strongly correlated and operate jointly to provide hope. Overall, these conceptualizations of hope refer to a personal hope, to the idea that the future will be better than the present for the individual, together with the belief that the person has the ability to act to reach what he/she cares about (Lopez, 2013).

Different studies showed how personal state hope is related to vocational identity, career decidedness, mastery goal orientation, and satisfaction for educational plans (Gilman et al., 2006; Kenny et al., 2010; Lodi et al., 2017). Moreover, as concerns the relation between hope and the investment in higher education, previous researches on students’ hope have found that it functions as a motivational trigger to achieve a positive outcome in the academic field; specifically, it is related to college achievement, academic engagement, academic success, adaptation to academic life, and academic self-efficacy (Gilman et al., 2006; Kenny et al., 2010; Bakker and Demerouti, 2014; Gallagher et al., 2017).

In addition to individual hope, over the last years, given the global changes and challenges and the necessity to act in order to promote a sustainable development, some forms of collective or social hope have been underlined (Watts et al., 2011; Anderson et al., 2016). Social hope refers to the fact that its subject is collective and not individual: *We* and not *I* can change the future. Anderson et al. (2016) underlined that people come together and collectively articulate goals that are referred to social goods to be achieved. People are motivated by the pursuit of goals that cannot be reached in an individual way.

A pilot study conducted by Di Maggio et al. (2018) aimed at examining the psychometric requisites of a questionnaire to assess state personal and social hope in adolescents, found a monofactorial structure (personal and social hope), and positively correlated with career adaptability and the tendency to consider systemic challenges to attain a sustainable development. Based on these findings, in this study, the questionnaire was used to consider both the more individual-centered aspects (state personal hope) and more societal-centered aspects (state social hope).

**Hypothesis 3.1.** State personal and social hope mediate the relationships between career adaptability, the tendency to consider systemic challenges to attain sustainable development, and investment in higher education (see Figure 1).

**Hypothesis 3.2.** State personal and social hope is related to the tendency to invest in higher education (see Figure 1).

## Materials and Methods

### Participants

Four hundred sixteen Italian high school students (*M*_*age*_ = 17.28 years, *SD* = 0.85), all residents in the northeast of Italy (Veneto region), were involved in this study. The sample was composed of 175 (42.1%) boys and 241 (57.9%) girls. Moreover, in line with the Italian high school population, 10.3% of the participants involved in the study were students of vocational schools, 40.3% of the participants were students of technical schools, and 49.4% were students of lyceums.

### Measures

*Career Adapt-Abilities Scale-Italian Form* (Soresi et al., 2012) was used to assess career adaptability. Specifically, this instrument is composed of 24 items saturated in four subscales, with six items each: concern (e.g., “Thinking about what my future will be like”; α = 0.80), control (e.g., “Taking responsibility for my actions”; α = 0.74), curiosity (e.g., “Observing different ways of doing things”; α = 0.77), and confidence (e.g., “Solving problems”; α = 0.85). The scale showed adequate internal consistency estimates and a coherent multidimensional structure. Moreover, this questionnaire has been used with Italian samples in different studies (e.g., Ginevra et al., 2016; Santilli et al., 2017). In this study, Cronbach’s alpha for four subscale were 0.84, 0.80, 0.80, and 0.85, and 0.93 for the total score.

*The future is around the corner*… *what will it hold for us? An instrument on UN’s goals for the inclusive and sustainable development* (Santilli et al., unpublished). This questionnaire allows to analyze the tendency to consider systemic challenges in order to attain a sustainable development. Specifically, with its 17 items, it refers to the 17 goals presented in the 2030 Agenda for Sustainable Development. Each participant is asked how much he/she thinks that every goal presented can affect his/her educational and career choices. An example of an item is the following: “In the future there will certainly still be much to do to ensure employment and decent work for all… How could this topic influence your educational and career choices? Adolescents are invited to express their views on a 5-point Likert scale (1 = *too little*, 5 = *very much*). Analyses carried out by Santilli et al. (unpublished) found that the questionnaire is a psychometrically valid and reliable measure. This instrument was used by Nota et al. (2018) in a cluster study with Italian adolescents. In this study, Cronbach’s alpha was 0.91.

*Hope for the Future* (Di Maggio et al., 2018). The instrument was used to analyze personal and social hope. This self-report measure is a 12-item monofactorial instrument. Examples of items are “In the future I will be happier than today” and “In the future the respect for human rights and equality among people will certainly be better.” Respondents indicated how much they agreed with each item using a 5-point scale (1 = *strongly disagree*, 5 = *strongly agree*). Previous analyses carried out by authors showed good psychometric properties for the instruments with an internal consistency estimate of 0.64. The questionnaire was used by authors to test the role of social and personal hope in education investment and school performance, with an Italian sample of adolescents. In this study, Cronbach’s alpha was 0.64.

*Tendency to Invest in Higher Education* was assessed using a factor of the questionnaire “Keeping pace with the times that will come” (Di Maggio et al., 2018). The factor, consisting of 11 items, assesses the tendency of adolescents to invest in their future on higher education. An example of item is: “I know that in the future I will have to spend some time on my higher education and this doesn’t frighten me.” Respondents indicated how much they agreed with each item using a 5-point scale (1 = *strongly disagree*, 5 = *strongly agree*). Previous analyses carried out by authors showed the good psychometric properties of the instruments with an internal consistency estimate of 0.82. The questionnaire was recently used to verify the relationship between courage, tendency to invest in higher education, and quality of life in a sample of Italian adolescents (Nota et al., 2019). In this study, Cronbach’s alpha was 0.85.

### Procedure

In this study, we used a multistage sampling method, as suggested by different authors (e.g., De Carlo and Robusto, 1996; Dudovskiy; 2018). Thanks to its high levels of flexibility, it can be considered a good method in terms of cost-effectiveness and time-effectiveness, in particular in primary data collection from geographically dispersed areas, when in the study there is the need to involve parts of the population with face-to-face contact. In this study, different high schools, randomly selected within the Veneto region (non-probability sampling), were asked to participate in a vocational career guidance project aimed at enabling students to think about their future. Once the subscriptions of the different high schools were gathered, the 11th and 12th-grade classes that could participate in the project were randomly selected. Within every selected class, the students were allowed to join the project through parental consent and voluntary participation. All the students of the selected classes decided to join the project.

Every participant (and their parents for adolescents under 18 years of age) was informed about the project goal, and all the gathered information was protected by professional confidentiality, following ethical procedures ruled by the Italian Ethical Principles of Psychologists. The questionnaires were administered in small groups of participants by a trained career counselor in a confidential classroom provided by the school. The assessment phase lasted approximately 45 min.

### Data Analysis

#### Preliminary Analysis

In order to test the hypothesized model, a number of preliminary analyses were performed: screening for missing data, indices of normality (e.g., skew and kurtosis), means, standard deviations, and correlations.

#### Mediational Analysis

A structural equation modeling (SEM), using the software Lisrel 8.7, was executed to test the hypothesized model. Specifically, a procedure based on two steps (Anderson and Gerbing, 1988; Barbaranelli and Ingoglia, 2013) was carried out. Specifically, in the first step, we tested the measurement model, describing the relationships between observed variables and the constructs (latent variables), and in the second step was the structural model, examining the relationships between the constructs hypothesized. Structural equation models with latent variables allow to better approximate theoretical constructs (that are not measurable) because they take into consideration the measurement error of the observed variables, specifying it within the model (Anderson and Gerbing, 1988; Little et al., 2002; Barbaranelli and Ingoglia, 2013).

#### Measurement Model

A covariance matrix with 11 measured variables as input data was used. The first factor loading for each latent variable was fixed to one as the default. All other factor loadings and paths among the four latent variables were freely estimated. To generate latent variables of career adaptability, tendency to consider systemic challenges to attain sustainable development, personal and social hope, and tendency to invest in higher education, the item parceling method was used, because it has been considered a better model fit in respect to using all items as indicators (Little et al., 2002). Specifically, for career adaptability, the internal-consistency approach was used (Kishton and Widaman, 1994). We created four parcels, given by the average of items each factor. For the tendency to consider systemic challenges to attain sustainable development, personal and social hope, and the tendency to invest in higher education, the item-to-construct balancing technique was used (Little et al., 2002). Specifically, we firstly analyzed the mono-dimensionality of these three measures using a principal axis factoring (PAF), and then we created three parcels for the tendency to consider systemic challenges to attain sustainable development and two parcels for personal and social hope and the tendency to invest in higher education, based on the magnitude of the factor loadings in the PAF.

To evaluate the goodness of fit of the model, the following indices were used: the root mean square error of approximation (RMSEA), the comparative fit index (CFI), the non-normed fit index (NNFI), and the standardized root mean square residual (SRMR). Acceptable model fit was defined by the following multiple cutoff values: RMSEA ≤ 0.06, CFI ≥ 0.95, NNFI ≥ 0.95, and SRMR ≤ 0.08.

#### Structural Model

In the second step, we tested the direct and indirect relationships from career adaptability and the tendency to consider systemic challenges to attain sustainable development to the tendency to invest in higher education, through the mediational role of state personal and social hope. The goodness of fit was evaluated through the same indices above indicated (RMSEA, CFI, NNFI, and SRMR).

Lastly, the significance of the indirect effects was examined, using the asymmetric confidence intervals test (MacKinnon et al., 2007).

## Results

### Preliminary Analysis

Missing data replacement (low than 1%) was performed using person mean replacement (Pigott, 2001). Skewness and kurtosis were found acceptable for all variables considered. Correlation analysis between the latent variables showed positive correlations with a magnitude from 0.15 to 0.38 (see Table 1), and the variance inflation factor (VIF) index for all variables (range VIFs = 1.18–1.04) was acceptable (lower than the recommended 5.0; Hair et al., 2011).

**TABLE 1:** Means, standard deviations, and correlations.

Study variables	*M*	SD	1	2	3	4
1. Tendency to consider systemic challenges in order to attain a sustainable.	2.87	0.76	–	0.152**	0.164**	0.156**
2. Career adaptability	3.82	0.57		–	0.302***	0.384***
3. State personal and social hope	3.42	0.53			–	0.376**
4. Investment in higher education	3.47	0.69				–

***Correlations were significant at p < 0.001. **Correlations were significant at p < 0.01.

### Measurement Model

The measurement model fit was satisfactory: χ^2^_(__38__)_ = 65.502, CFI = 0.990, RMSEA = 0.042, and SRMR = 0.031. All factor loadings were significant (*p* < 0.001) and ranged from 0.56 to 1.00.

### Structural Model

**FIGURE 2:**
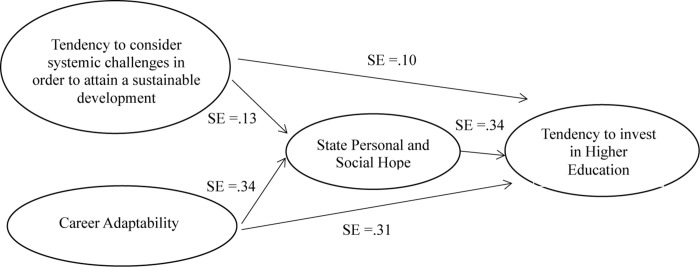
Significant standardized parameter estimates in the fully mediated model. SE, standardized parameter estimates. Solid lines indicate statistically significant effects with *p* < 0.05.

The hypothesized structural model (see Figure 2) had good fit indices: χ^2^_(__38__)_ = 65.502, CFI = 0.990, RMSEA = 0.042, and SRMR = 0.031. Specifically, the results showed that the relationships between career adaptability and state personal and social hope (β = 0.34; *t* = 4.81; *p* < 0.05) and between career adaptability and the tendency to invest in higher education in a direct way (β = 0.31; *t* = 5.87; *p* < 0.05) were significant. Moreover, it was found that the relationships between the tendency to consider systemic challenges in order to attain a sustainable development and state personal and social hope (β = 0.13; *t* = 2.36; *p* < 0.05) and between the tendency to consider systemic challenges in order to attain a sustainable development and the tendency to invest in higher education (β = 0.10; *t* = 2.24; *p* < 0.05) were significant. Lastly, the relationship between state personal and social hope state and the tendency to invest in higher education was significant (β = 0.34; *t* = 6.12; *p* < 0.05). Overall, *R*^2^ for state personal and social hope state was 0.867, accounting for 87% of the variance; and for the tendency to invest in higher education, it was 0.791, accounting for 79% of the variance.

Regarding the significance of the indirect effects (see Table 2), it was found that the 95% confidence intervals for the indirect effect between the tendency to consider systemic challenges in order to attain sustainable development and the tendency to invest in higher education ranged from 0.007 to 0.086. Instead, the indirect effect between career adaptability and the tendency to invest in education ranged from 0.077 to 0.227. These values did not include a zero, suggesting that these indirect effects are supported.

**TABLE 2:** Unstandardized and standardized direct and indirect effects for fully mediated model.

	Direct effect			Indirect effect			Total effect			
Predictor	US	SE^a^	S	US	SE^a^	S	US	SE^a^	S	95% CI^b^
Tendency to consider systemic challenges	0.10	0.04*	0.10	0.04	0.02*	0.05	0.14	0.05*	0.15	0.007–0.086
Career adaptability	0.38	0.07*	0.31	0.15	0.04*	0.12	0.53	0.06*	0.42	0.077–0.227

US, unstandardized; S, standardized; CI, confidence interval. ^a^Standard error for the maximum likelihood estimate. ^b^Significance of mediated effect with PRODCLIN. *Statistically significant effects with p < 0.05.

## Discussion

Based on the career construction adaptation model (Savickas and Porfeli, 2012) and on recent directions from the perspective of sustainable and inclusive career guidance (e.g., Guichard, 2018), the goal of this study was to examine the relationship between career adaptability and the tendency to consider systemic challenges to attain sustainable development (as adaptability resources), state personal and social hope (as adapting response), and their role in affecting the tendency to invest in higher education (as adaptation result).

Results suggest that career adaptability was indirectly related to the investment in higher education, through state personal and social hope. Specifically, these results were in line with Rudolph et al.’s (2017) meta-analysis and different other studies (e.g., Ginevra et al., 2016; Santilli et al., 2017). Based on life design approach and the career construction adaptation model (Savickas and Porfeli, 2012), these results showed how career adaptability predicted investment in higher education, directly and indirectly through hope. This finding is also in line with different studies (Gilman et al., 2006; Kenny et al., 2010; Gallagher et al., 2017) that showed a positive connection between hope for the future and different educational outcomes such as academic achievement, engagement, success, and self-efficacy.

These relations suggest that the consideration of the self as someone able to think about and construct his/her future career intentions, to be curious, to explore, and to assume responsibilities may favor in adolescents the possibility to detect different future options. This can act as a motivational factor leading adolescents to imagine personal and social goals and to sustain the effort needed to achieve goals and wishes for a better future, and also together with other people. Being able to imagine those goals and ways to pursue them could, therefore, lead adolescents to understand the importance of investing in higher education and to be more motivated in taking educational paths for a better personal and collective future (Akkermans et al., 2018).

The results of this study showed also direct and indirect relationships, through the mediational role of state personal and social hope, between the tendency to consider systemic challenges to attain sustainable development, and the tendency to invest in higher education. These findings confirm other studies (e.g., Diemer et al., 2006; Duffy et al., 2016; Gallagher et al., 2017) that showed a positive relationship between the ability to systematically analyze current social conditions, hope for the future, educational outcomes such as career choices, and continuing a career path. To explain these results, we need to consider the difficulties of the labor market in terms of challenges connected to an inclusive and sustainable development rather than “events” or unpleasant characteristics that cannot be modified. Considering difficulties and challenges in this way could motivate adolescents to perform agentive behaviors connected to the identification of personal and social future goals (personal and social hope), and to the realization of useful paths for the pursuit of those goals, such as investing in higher education and reconsidering its benefits and costs (Abbiati and Barone, 2017).

The results of this study also support the mediational role of state personal and social hope. State hope reflects individuals’ motivation and pushes toward important goals for the person. It is also a useful ability to collectively articulate goals related to social topics such as the respect for human rights and the elimination of any form of discrimination (Anderson et al., 2016). To pursue these social goals, everyone’s shared responsibilities and commitment are required (Anderson et al., 2016). The influence of hope on the investment in higher education could be due to the fact that the union of individual and collective hope can motivate adolescents to assume higher responsibilities and commitment toward a better future. When this happens, the future can appear to be modifiable. These feelings can make adolescents feel stronger in front of future challenges and can lead them to feel more motivated in change-oriented actions (Bakker and Demerouti, 2014).

Finally, the results of this study showed that career adaptability directly predicted the tendency to invest in higher education in a deeper way than the tendency to consider systemic challenges to attain sustainable development. This result could be explained by the relevance of four career adaptability resources in future career planning in the last decades, which are characterized by uncertainty and precariousness. However, in this study, the tendency to consider systemic challenges to attain sustainable development has been detected as an important resource to consider. Lastly, personal and social hope had a significant similar mediational role between the two predictors and the outcome, although less strong than direct relationships, suggesting that it is equally relevant in the relationships between career adaptability, the tendency to consider systemic challenges to attain sustainable development, and tendency to invest in higher education, as it helps to envision future personal and social goals and plan strategies to achieve them.

### The Theoretical Contribution of the Study

From a theoretical perspective, this study contributes to extending the previous results described by the career construction adaptation model, considering the recent directions to contribute to inclusive and sustainable future contexts. For the first time, in this study, attention has been given simultaneously to the individual and to social dimensions to explain how much adolescents tend to invest in higher education. Firstly, in addition to career adaptability, denoting individual agency to proactively manage career transitions, we analyzed the tendency to consider systemic challenges to attain sustainable development. The results obtained underline how the tendency to consider said challenges can acquire a value in the reflections that young people do in favor of their future. This tendency to consider systemic challenges to attain sustainable development seems to work alongside career adaptability as a further adapting resource, in which presence can foster the design of a career future beyond the negative conditions that people experience. Secondly, the results obtained in this study showed the relevance of considering both state personal and social hope in career construction. Social hope could lead adolescents to consider the future in terms of shared responsibilities and collective commitment that could help them to better cope with the challenges connected to the future (Watts et al., 2011; Anderson et al., 2016).

### The Practical Contribution of the Study

From a practical perspective, this study outlines a preventive career education intervention in high schools. In particular, this study considers the relevance of the tendency to invest in higher education in producing graduates able to find new, complex, and innovative solutions to create a future society able to provide well-being and satisfaction (Peterson and Helms, 2014). The results of our study suggest implementing in schools preventive actions supporting career adaptability resources, for example, career interventions for adolescents based on the life design approach developed by Nota et al. (2016). These interventions can be implemented with high school students in small groups by trained teachers and career practitioners. Moreover, we considered the relevance of the tendency to explore systemic challenges to attain sustainable development in determining a hopeful attitude toward one’s future and toward the society and a higher degree of tendency to invest in higher education. As a consequence, career interventions in schools should implement the tendency to reflect on nowadays and future challenges to be able to start building inclusive and sustainable societies. Specifically, it could be useful to design activities to encourage young people to reflect about the future, considering which study course to choose and what professional contribution to give, to play their part in achieving by 2030 at least some of the 17 goals that the UN has presented to the whole world.

### Limitations of the Study

There are considerable limitations to this research that can be helpful for future studies. To begin with, even if a structural equation method was performed to examine “causal” hypotheses, data collected were cross-sectional and, as a consequence, could not bring evidence of actual causation. In future studies, it would be better to use a structural equation longitudinal method. In the second place, self-reported measures were administered to assess the dimensions of this study. Future research should take into consideration different methods to reduce the influence of self-report bias. Lastly, it is important to recognize that multistage sampling is not as effective as true random sampling; nonetheless, it focuses on specific disadvantages connected with true random sampling such as being overly expensive and time-consuming.

## Conclusion

This study considered the new global challenges and the need to move toward career planning processes and career development able to contribute to the construction of inclusive and sustainable future contexts. It provided a contribution to literature about the role of career adaptability and the tendency to consider systemic challenges to attain sustainable development as relevant adaptability resources on adolescents’ tendency to invest in higher education. The study also highlighted the mediational role of combining state personal and social hope in favoring higher responsibility and commitment toward a better future for everybody and, therefore, in promoting more investment in higher education to find new, complex, and innovative solutions to guarantee an inclusive and sustainable future.

## Data Availability Statement

All datasets generated for this study are included in the article/supplementary material, further inquiries can be directed to the corresponding author.

## Ethics Statement

Ethical review and approval was not required for the study on human participants in accordance with the local legislation and institutional requirements. Written informed consent to participate in this study was provided by the participants’ legal guardian/next of kin.

## Author Contributions

All authors contributed to the research effort reported in the manuscript.

## Conflict of Interest

The authors declare that the research was conducted in the absence of any commercial or financial relationships that could be construed as a potential conflict of interest.
